# Supplementary material for: Systemic cisplatin increases the number of patients showing positive off-frequency masking audiometry
Source: PLoS One. 2023 Jul 6;18(7):e0287400. doi: 10.1371/journal.pone.0287400 (PMC10325046; doi:10.1371/journal.pone.0287400)
Supplement: Attachment — Submitted filename: review plos 1.docx [file pone.0287400.s003.docx]

I would like to thank the authors for their submission and allowing me to review their work.

This is an interesting study on an important topic. However, I would be grateful if you could add further explanations and changes on the following points:

**1) Page 2, line 17**

Please specify the age range, gender and mean age (± standard deviation) of the study population.

**2) Page 2, line 17**

Where was the study conducted?

**3) Page 6, line 93**

Please add ± standard deviation.

**4) Page 6, line 94**

Please add more specific demographic and clinical information about the study participants.

**5) Page 7, line 109**

Where were the audiological tests performed?

**6) Page 15, line 253**

The “limitations section” should be expanded (e.g. limited number of participants, lack of specific tests such as auditory brainstem responses,…).

**7) Page 16, line 273**

Which are the future prospects of this study?

**8)** I would also suggest a review of the English language due to some grammar and phrasing mistakes.
